# M2 Macrophages promote IL-33 expression, ILC2 expansion and mucous metaplasia in response to early life rhinovirus infections

**DOI:** 10.3389/fimmu.2022.952509

**Published:** 2022-08-12

**Authors:** Mingyuan Han, Haley A. Breckenridge, Shiuhyang Kuo, Shilpi Singh, Adam G. Goldsmith, Yiran Li, Jordan E. Kreger, J. Kelley Bentley, Marc B. Hershenson

**Affiliations:** ^1^ Department of Pediatrics, University of Michigan Medical School, Ann Arbor, MI, United States; ^2^ Department of Molecular and Integrative Physiology, University of Michigan Medical School, Ann Arbor, MI, United States

**Keywords:** IL-33, Rhinovirus, neonate, ILC2, M2 macrophage

## Abstract

Wheezing-associated rhinovirus (RV) infections are associated with asthma development. We have shown that infection of immature mice with RV induces type 2 cytokine production and mucous metaplasia which is dependent on IL-33 and type 2 innate lymphoid cells (ILC2s) and intensified by a second heterologous RV infection. We hypothesize that M2a macrophages are required for the exaggerated inflammation and mucous metaplasia in response to heterologous RV infection. Wild-type C57Bl/6J mice and LysM^Cre^ IL4Rα KO mice lacking M2a macrophages were treated as follows: (1) sham infection on day 6 of life plus sham on day 13 of life, (2) RV-A1B on day 6 plus sham on day 13, (3) sham on day 6 and RV-A2 on day 13, or (4) RV-A1B on day 6 and RV-A2 on day 13. Lungs were harvested one or seven days after the second infection. Wild-type mice infected with RV-A1B at day 6 showed an increased number of *Arg1-* and *Retnla*-expressing lung macrophages, indicative of M2a polarization. Compared to wild-type mice infected with RV on day 6 and 13 of life, the lungs of LysM^Cre^ IL4Rα KO mice undergoing heterologous RV infection showed decreased protein abundance of the epithelial-derived innate cytokines IL-33, IL-25 and TSLP, decreased ILC2s, decreased mRNA expression of IL-13 and IL-5, and decreased PAS staining. Finally, mRNA analysis and immunofluorescence microscopy of double-infected LysM^Cre^ IL4Rα KO mice showed reduced airway epithelial cell IL-33 expression, and treatment with IL-33 restored the exaggerated muco-inflammatory phenotype.

**Conclusion:**

Early-life RV infection alters the macrophage response to subsequent heterologous infection, permitting enhanced IL-33 expression, ILC2 expansion and intensified airway inflammation and mucous metaplasia.

## Introduction

Early-life wheezing-associated respiratory tract infections by rhinovirus (RV) and are considered risk factors for asthma development (1–3). Children are infected with many different RV strains, with infants having 6-10 distinct RV infections per year (4). However, it is unclear whether early-life RV infection, in combination with other factors such as allergen sensitization, genetic factors or microbiome, causes asthma, or whether wheezing-associated RV infections simply identify children who are already predisposed to asthma development. To test whether early-life viral infection could contribute to asthma development, we established a mouse model of early-life RV infection. RV infection of 6 day-old mice, but not mature mice, induced expansion of IL-13-producing type 2 innate lymphoid cells (ILC2s), mucous metaplasia and airway hyperresponsiveness (5, 6). Immature mice are also deficient in type 1 (IFN-γ and IL-12) antiviral responses. Subsequent studies showed that IFN-γ blocks type 2 airway inflammation and mucous metaplasia by interfering with ILC2 function (7), that ILC2s are required and sufficient for the (8), and that IL-33 was required and sufficient for the expansion of ILC2s (9). More recently, we found that early-life RV infection alters the response to subsequent heterologous infection, inducing intensified type 2 inflammation, mucous metaplasia and airways hyperresponsiveness which were dependent on ILC2s (10). In these experiments, we employed RV-A1B and RV-A2, minor group RVs that attach to proteins of the low-density lipoprotein receptor (LDL-R) family on the cell surface. LDL-R family proteins are highly conserved between human and mouse, allowing a modest amount of viral replication in murine epithelial cells (11, 12), in contrast to major group RVs which bind intercellular adhesion molecule-1. Also, RV-A1B does not induce specific immunity to RV-A2, even though they are from the same species (13, 14).

In contrast to M1-like macrophages that are stimulated by IFN-γ and lipopolysaccharide and promote cytotoxicity, M2a macrophages are classically stimulated by IL-4, IL-13 and IL-33 and promote cell growth and tissue repair. For example, M2a-like macrophages hold increased arginase activity, thereby downregulating nitric oxide production and increasing the production of ornithine, which is required for cell proliferation and collagen biosynthesis (15). Recent studies demonstrate a reciprocal relationship between IL-13-producing ILC2s and M2a alternatively-activated macrophages. Accordingly, IL-13 production by ILC2s activates M2a macrophages required for lung immunity against hookworms (16). In addition, there is a positive correlation between numbers of ILC2s and M2a-polarized macrophages in asthmatic sputum, and co-culture of ILC2s with alveolar macrophages induced expression of M2 macrophage-related genes (17). Conversely, infection with influenza and RSV infection each stimulate IL-33 production from lung macrophages, which in turn activates ILC2s (18–20).

In the present study, we hypothesized that early-life RV infection polarizes airway macrophages to M2a macrophages which are in turn required for the exaggerated type 2 inflammation and mucous metaplasia in response to a second, heterologous RV infection. To accomplish this, wild-type C57Bl/6J and LysM^Cre^ IL4Rα KO mice lacking M2a macrophages (21) were infected with RV-A1B and RV-A2 on days 6 and 13 of life, respectively. We found that, compared to wild type mice, LysM^Cre^ IL4Rα KO mice showed decreased type 2 inflammation, mucous metaplasia and innate cytokine expression.

## Methods

### Animals

All animal usage was approved by the Institutional Animal Care and Use Committee and followed guidelines set forth in the Principles of Laboratory Animal Care from the National Society for Medical Research. C57BL/6J mice were purchased from Jackson Laboratory (Bar Harbor, ME) and bred in house in pathogen-free facility within the Unit for Laboratory Animal Medicine at the University of Michigan. Macrophage/neutrophil-specific IL-4 receptor alpha-deficient (LysM^Cre^ IL-4Rα KO) mice were a gift from Frank Brombacher, International Centre for Genetic Engineering and Biotechnology, Cape Town, South Africa (21).

### Generation of RV-A1B and RV-A2

RV-A1B and RV-A2 (ATCC, Manassas, VA), minor group viruses that infect mouse cells (11), were partially purified from infected HeLa cell lysates by means of ultrafiltration with a 100-kDa cutoff filter and titered by using a plaque assay as described previously (12, 22). Intact virus does not go through the filter and is concentrated. Similarly concentrated and purified HeLa cell lysates were used for sham infection.

### RV infections

Mice were inoculated with 15 µl of 1 x 10^8^ plaque-forming units (PFU) or sham HeLa cell lysate through the intranasal route under Forane anesthesia. Mice were treated on day 6 of life with RV-A1B and day 13 of life with RV-A2 as follows: 1) day 6 sham + day 13 sham; 2) day 6 RV-A1B + day 13 sham; 3) day 6 sham + day 13 RV-A2; and 4) day 6 RV-A1B + day 13 RV-A2. We chose six-day-old mice for the first RV infection because these mice demonstrated an immature type 2 inflammatory response to infection (characterized by high IL-13 and low IFN-γ) (6). In contrast, older mice showed a relatively mature antiviral response characterized by low IL-13 and high IFN-γ. Immature animals of both sexes were used, as our previous study showed no differences in the response to RV infection between groups (10). Selected LysM^Cre^ IL-4Rα mice were treated with either PBS or 0.1 µg of recombinant murine IL-33 in 15 µL PBS (PeproTech, Rocky Hill, NJ) intranasally on day 13 one hour before RV-A2 infection.

Our previous studies showed differential kinetics for the various outcomes of viral infection. mRNA expression of IL-1β, CXCL-1, TNF-α, IFN-γ and IL-33 peaked one day after infection, whereas expression of type 2 cytokines, IL-25, TSLP, mucin genes and PAS staining peak seven days after infection. We therefore terminated the experiments on day of life 14 (one day after the second infection with sham or RV-A2) or day of life 20 (seven days after the second infection).

### Real-time quantitative PCR

Lung RNA was extracted with Trizol (Invitrogen, Carlsbad, CA) and isolated using an RNAeasy kit (Qiagen, Germantown, MD). cDNA was synthesized from 2 μg of RNA using high capacity cDNA synthases kit (Applied Biosystems, Foster City, CA) and subjected to quantitative real-time PCR using specific primers (**Table 1**) for mRNA. The level of gene expression for each sample was normalized to GAPDH. To quantify viral copies, qPCR for positive-strand viral RNA was conducted using RV-specific primers and probes (forward primer: 5’-GTGAAGAGCCSCRTGTGCT-3’; reverse primer: 5’-GCTSCAGGGTTAAGGTTAGCC-3’; probe: 5’-FAM-TGAGTCCTCCGGCCCCTGAATG-TAMRA-3’).

**Table 1:** Primer sequences for real-time PCR.

Primers	Sequence
MouseIFN-γ (Forward)	5’-TGGCTGTTTCTGGCTGTTAC-3’
MouseIFN-γ (Reverse)	5’-TCCACATCTATGCCACTTGAGTT-3’
MouseIL-1β (Forward)	5’-TGGCAGCTACCTGTGTCTTTC-3’
MouseIL-1β (Reverse)	5’-GGATGGGCTCTTCTTCAAAGATG-3’
MouseIL-5(Forward)	5’-CTCTGTTGACAAGCAATGAGACG-3’
MouseIL-5 (Reverse)	5’-TCTTCAGTATGTCTAGCCCCTG-3’
MouseIL-13 (Forward)	5’-CCTGGCTCTTGCTTGCCTT-3’
MouseIL-13 (Reverse)	5’-GGTCTTGTGTGATGTTGCTCA-3’
MouseIL-25 (Forward)	5’-ACAGGGACTTGAATCGGGTC-3’
MouseIL-25 (Reverse)	5’-TGGTAAAGTGGGACGGAGTTG-3’
MouseIL-33 (Forward)	5’-GGCTGCATGCCAACGACAAGG-3’
MouseIL-33(Reverse)	5’-AAGGCCTGTTCCGGAGGCGA-3’
MouseMuc5ac (Forward)	5’AAAGACACCAGTAGTCACTCAGCAA-3’
MouseMuc5ac (Reverse)	5’-GGTTTGACACTGACTTCCCAG-3’
MouseClca1 (Forward)	5’-CTGTCTTCCTCTTGATCCTCCA-3’
MouseClca1 (Reverse)	5’-CGTGGTCTATGGCGATGACG-3’
MouseCXCL-1 (Forward)	5’-TGCACCCAAACCGAAGAAGTCAT-3’
MouseCXCL-1 (Reverse)	5’- CAAGGGAGCTTCAGGGTCAAG-3’
MouseTNF-α (Forward)	5’-GCAGGTTCTGTCCCTTTCAG-3’
MouseTNF-α (Reverse)	5’-GTCGCGGATCATGCTTTCTG-3’
MouseArg-1 (Forward)	5’-AAGAATGGAAGAGTCAGTGTGG-3’
MouseArg-1(Reverse)	5’-GGGAGTGTTGATGTCAGTGTG-3’
MouseRetnla (Forward)	5’-AGGAACTTCTTGCCAATCCAG-3’
MouseRetnla (Reverse)	5’-AGTCAACGAGTAAGCACAGG-3’

### Lung histology and immunofluorescence

Lungs were fixed with 10% formaldehyde overnight and paraffin embedded. Blocks were sectioned at 500 μm intervals at a thickness of 5 μm, and each section was deparaffinized, hydrated and stained. To visualize mucus, sections were stained with periodic acid-Schiff (PAS), which labels neutral mucins and glycogen ( Sigma-Aldrich, St. Louis, MO). PAS staining in the airway epithelium were quantified by NIH ImageJ software (Bethesda, MD). Six separate mouse lungs of either wildtype or LysM^Cre^ IL-4Rα mice from each of the four conditions were processed for sectioning. Two-to three separate airways of similar size from each lung were chosen for analysis. PAS expression was calculated as the fraction of PAS-positive epithelium compared with the total basement membrane length. Other lung sections were stained with 4′,6-diamidino-2-phenylindole (DAPI) and anti-mouse IL-33 antibody (clone 396118; R&D system, Minneapolis, MN) followed by Alexa Fluor™ 555 anti-Rat-IgG secondary antibody (ThermoFisher Scientific, Waltham, MA). Images were visualized using an Olympus IX71 microscope (Center Valley, PA) with appropriate filters.

### Flow cytometric analysis

Lungs were perfused with PBS containing 0.5 mM EDTA and minced and digested with Liberase TM (100 µg/ml; Roche, Indianapolis, IN), together with collagenase XI (250 µg/ml), hyaluronidase 1a (1 mg/ml), and DNase I (200 µg/ml; Sigma, St. Louis, MO) for 1 h at room temperature. Cells were filtered and washed with RBC lysis buffer (BD Biosciences, San Jose, CA). Nonspecific binding was blocked by 1% fetal bovine serum with 1% LPS-free bovine serum albumin in DMEM, and 5 μg rat anti-mouse CD16/32 (Biolegend) was added. To identify ILC2s, cells were stained with FITC-conjugated antibodies for lineage markers CD3ϵ, TCRβ, B220/CD45R, Ter-119, Gr-1/Ly-6G/Ly-6C, CD11b (Biolegend), CD11c (Biolegend), TCRβ (Biolegend), F4/80 (Biolegend), and FcϵRIα (Biolegend), and anti-CD127-allophycocyanin (APC; eBioscience), anti-ST2-phycoerythrin(PE)-Cyanine (Cy)7 (Biolegend), as described (23). Conjugated IgGs and combined fluorescence minus one (FMO) controls were used for each probe. To sort lung epithelial cells and macrophages, cells were stained with anti-CD45-PE-Cy7 (Biolegend), anti-F4/80-FITC, and anti-EpCAM-APC (Biolegend). To determine lung macrophages polarization, cells were stained with anti-F4/80-FITC (Biolegend), anti-CD11c-PE (Biolegend). After staining for cell surface antigens, dead cells were stained with DAPI for flow sorting with live cells or Pac-Blue Live/Dead fixable dead staining dye for further staining with anti-Arginase-1 antibody (eBioscience). For Arginase-1 staining, cells were fixed and permeabilized using permeabilization buffer (eBioscience) and stained with APC-labeled anti-Arginase-1 (eBioscience). Cells were subjected to flow cytometry on a LSR Fortessa (BD Biosciences), or sorted on a MA900 cell sorted (Sony Biotechnology, San Jose, CA). Sorted lung epithelial cells or macrophages were dissolved in RLT lysis buffer from RNAeasy kit (Qiagen) for subsequent PCR analysis. Data were analyzed using FlowJo software (Tree Star, Ashland, OR).

### Measurement of lung cytokine levels

Lung IL-13, CXCL1, TNF-α, IFN-γ, IL-25, IL-33 and TSLP (Thermo Fisher Scientific) were measured by ELISA. ELISA data were analyzed by BioTek Gen5 software (Winooski, VT) and normalized for total lung protein concentration. Total lung protein concentration was measured by BCA protein assay (Thermo Fisher Scientific).

### Data analysis

All data were represented as mean ± standard deviation (SD). Data from each group were tested for normality using the Shapiro-Wilk test. For normally distributed data, statistical significance was assessed by one-way analysis of variance. Group differences were pinpointed by the Tukey multiple comparison test. If normality was not present, statistical significance was determined using the Kruskal Wallis test and differences between groups, if present, were pinpointed using Dunn’s multiple comparison test.

## Results

### Heterologous RV infection polarize airway macrophages toward an M2a-like phenotype

**Figure 1:**
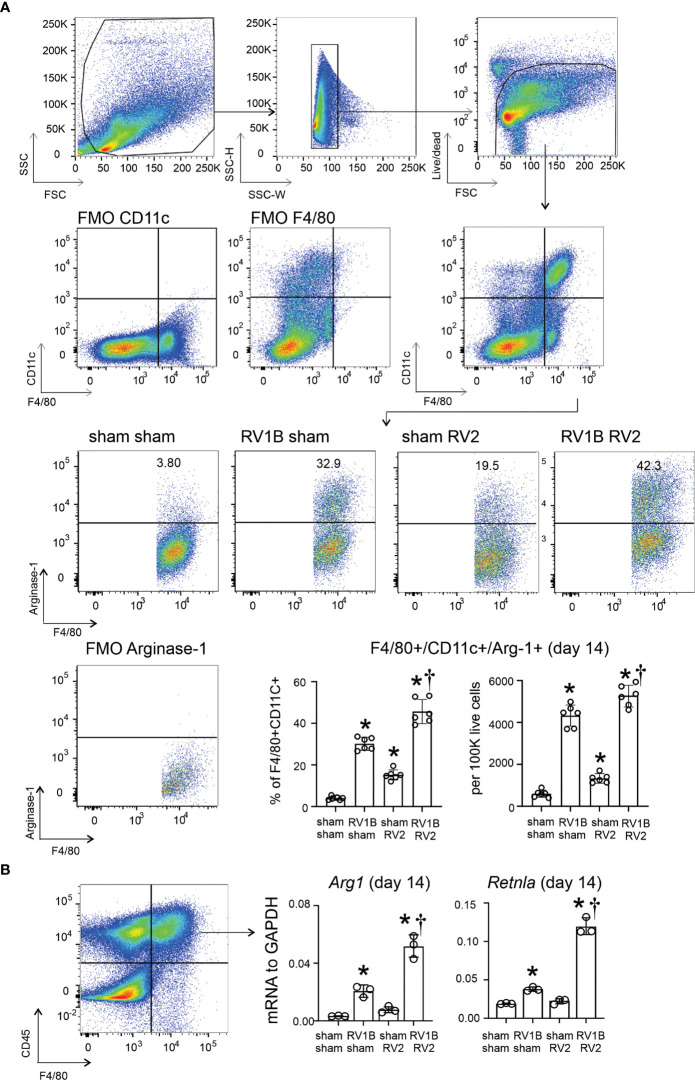
Differential expansion of F4/80+CD11c+arginase-1+ M2a-polarized macrophages in wild-type immature mice following heterologous RV infection. Six-day old wild-type mice were inoculated with sham or RV-A1B on day 6 of life and sham or RV-A2 on day 13 of life. **(A)** On day 14, lungs were harvested, digested with liberase TM, collagenase XI, hyaluronidase 1a, and DNase I, and stained with anti-F4/80, anti-CD11c, anti-arginase-1, and PacBlue (for dead cells). Cells were washed, fixed, and processed for flow cytometry. The panel shows flow cytometry analysis of live F4/80+ CD11c+ arginase-1+ macrophages from the four groups. FMO controls for F4/80, CD11c and arginase are also shown. Data shown are mean ± SD; n=6 per group from two different experiments; *different from sham + sham, P < 0.05 by one-way ANOVA; †different from RV-A1B + sham, P < 0.05 by one-way ANOVA. **(B)** Lungs were collected on day 14 of life (1 day post RV-A2 or sham infection) from wild-type immature mice inoculated with sham or RV-A1B on day 6 of life and sham or RV-A2 on day 13 of life. Cell suspensions were sorted for CD45+ F4/80+ macrophages. The cell pellet was collected for mRNA expression by quantitative PCR (N=6/group, lungs from two mice were combined for each measurement). Data shown are mean ± SD; *different from sham+sham, *P* < 0.05 by one-way ANOVA; †different from RV-A1B+sham, *P* < 0.05 by one-way ANOVA.

We previously found that RV-A1B infection of six-day-old mice, but not mature mice, induced type 2 inflammation and mucous metaplasia which is dependent on IL-13-producing ILC2s and epithelial-derived innate cytokines (6). In the present study, we hypothesized that early-life RV infection polarizes airway macrophages to M2a macrophages which are required for exaggerated type 2 inflammation and mucous metaplasia in response to a second heterologous RV infection. M2a-like polarization of lung macrophages was determined by measuring the number of F4/80+/CD11c+/arginase-1+ lung cells by flow cytometry. RV-A1B infection on day 6 of life increased lung F4/80+/CD11c+/arginase-1+ cells compared to sham infection (**Figure 1A**). Heterologous infection with RV-A1B and RV-A2 increased F4/80+/CD11c+/arginase-1+ cells compared to RV-A1B alone. Finally, compared to sham-infected mice, sorted F4/80+/CD45+ cells from RV-A1B-infected mice showed higher expression of mRNAs encoding the M2a markers arginase-1 (*Arg1*) and Resistin-like molecule α (*Retnla*) (**Figure 1B**). *Arg1* and *Retnla* were further increased in sorted macrophages from mice infected with RV-A1B and RV-A2 compared to RV-A1B alone (**Figure 1B**).

### LysM^Cre^ IL-4Rα KO attenuated heterologous RV infection-induced type 2 inflammation and innate cytokine expression

**Figure 2:**
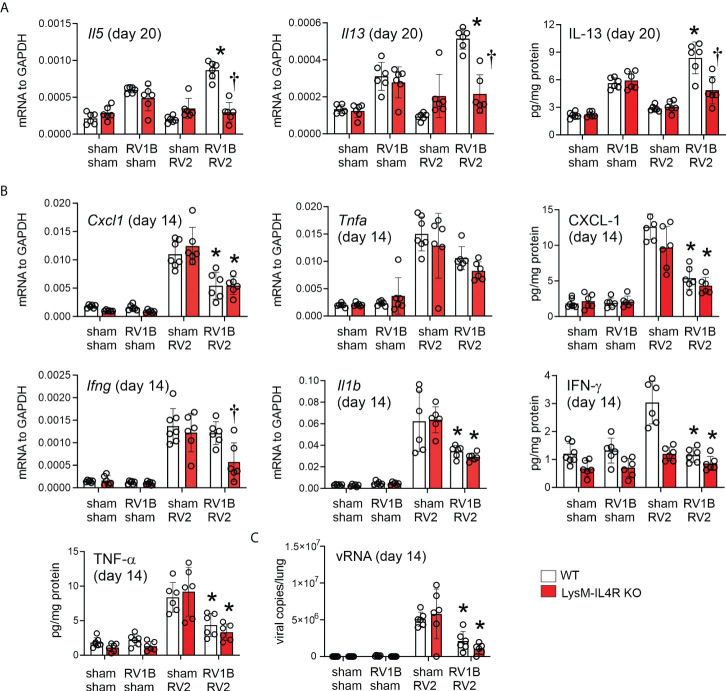
LysM^Cre^ IL-4Rα KO attenuates heterologous RV infection-induced type 2 cytokine expression. Six-day old immature wild-type and LysM^Cre^ IL-4Rα KO mice were inoculated with sham or RV-A1B on day 6 and sham or RV-A2 on day 13 of life. **(A)** mRNA and protein expression of the type 2 cytokines IL-5 and IL-13. Lungs were harvested for qPCR or ELISA seven days after secondary sham or RV-A2 infection. (N =6 from two different experiments, mean ± SD; *different from wildtype sham + RV-A1B, P < 0.05 by one-way ANOVA; †different from wild-type RV-A1B + RV-A2, P < 0.05 by one-way ANOVA.) **(B)** mRNA and protein expression of the pro-inflammatory cytokines IL-1β, TNF-α, CXCL-1, and IFN-γ. Lungs were harvested for qPCR or ELISA one day after secondary sham or RV-A2 infection. (N =6 from two different experiments, mean ± SD; *different from wild-type sham + RV-A2, P < 0.05 by one-way ANOVA; †different from wild-type RV-A1B + RV-A2, P < 0.05 by one-way ANOVA. **(C)** Whole lung RV positive-strand RNA one day after secondary infection with sham or RV-A2. (N=6 from two different experiments, mean ± SD; different from sham + RV-A2, P < 0.05 by one-way ANOVA.

To determine the effects of M2a macrophages on the subsequent heterologous infection, wild-type C57BL/6J and LysM^Cre^ IL4Ra KO mice lacking M2a macrophages were infected with RV-A1B (or sham) and RV-A2 (or sham) on days 6 and 13 of life, respectively. Consistent with previous findings, heterologous RV infection of wild-type mice exaggerated mRNA expression of the type 2 cytokines IL-5 and IL-13 compared to mice infected with RV-A1B alone (**Figure 2A**). mRNA expression was measured 7 days after secondary infection (sham or RV-A2). Double-infected LysM^Cre^ IL-4Rα KO mice showed significantly reduced *Il5* and *Il13*, as well as IL-13 protein. In contrast, mice infected with RV-A1B alone did not show reduced type 2 cytokine expression.

In contrast, compared to mice infected with RV-A2 alone, mice infected with RV-A1B prior to RV-A2 showed reduced mRNA and protein expression of IL-1β, TNF-α and CXCL1 measured one day after secondary infection (**Figure 2B**), consistent with the notion that early life (day 6) rhinovirus infection modulates the immune response towards the subsequent (day 13) infection, skewing it towards a type 2 response and away from a type 1 response. IFN-γ in response to double infection was reduced in the LysM^Cre^ IL-4Rα KO.

Finally, viral copy number was measured 1 day after secondary infection. Mice infected with RV-A1B prior to RV-A2 showed significantly reduced viral copies compared to mice infected with RV-A2 alone (**Figure 2C**). We hypothesized that IFN production after the first infection prevents replication of the second virus and decreases viral copy number. We previously found that, in immature mice, IFN-λ is sustained seven days after the infection (5). We therefore examined IFN-λ levels one day after secondary RV-A2 or sham infection. Mice undergoing RV-A1B infection on day 6 and sham infection on day 13 tended to show persistent IFN- λ one day after sham infection and double-infected mice tended to show a further increase in type III interferon.

### LysM^Cre^ IL-4Rα KO blocked heterologous RV infection-exaggerated innate cytokine expression

**Figure 3:**
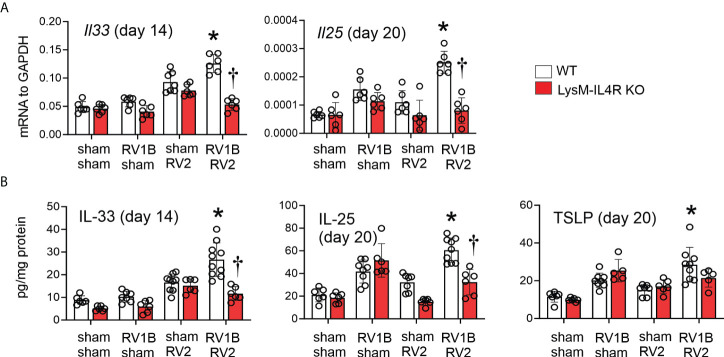
LysM^Cre^ IL-4Rα KO attenuated heterologous RV infection-enhanced innate cytokine expression. **(A, B)** Six-day old immature wild-type and LysM^Cre^ IL-4Rα KO mice were inoculated with sham or RV-A1B on day 6 and sham or RV-A2 on day 13 of life. Lungs were harvested for qPCR **(A)** or ELISA **(B)** one day post sham or RV-A2 secondary infection for IL-33 and seven days after sham or RV-A2 infection for IL-25 and TSLP expression. (N=6-10 from two different experiments, mean ± SD; *different from RV-A1B + sham (IL-25 and TSLP) or wild-type sham + RV-A2 (IL-33), P < 0.05 by one-way ANOVA; †different from wild-type RV-A1B+RV-A2, P < 0.05 by one-way ANOVA.

Our previous studies showed differential kinetics for IL-33, IL-25, and TSLP expression in RV-A1B infected immature mice. IL-33 expression peaked one day post-infection, whereas IL-25 and TSLP were elevated two days after infection and peaked on day 7 (9). Mice undergoing RV-A1B infection alone showed increased mRNA and protein expression of IL-25 (seven days after secondary infection) and IL-33 (one day after secondary infection) compared to sham-infected mice, and heterologous infection with RV-A2 further increased innate cytokine expression (**Figure 3**). A similar pattern was observed for TSLP protein (we previously found TSLP was not transcriptionally regulated). Compared to wild type mice, double-infected LysM^Cre^ IL-4Rα KO showed reduced IL-25, IL-33 and TSLP expression (**Figure 3**). Again, cytokine expression of mice infected with RV-A1B alone was not affected by the LysM^Cre^ IL-4Rα KO.

### LysM^Cre^ IL-4Rα KO reduced heterologous RV infection-induced ILC2 expansion

**Figure 4:**
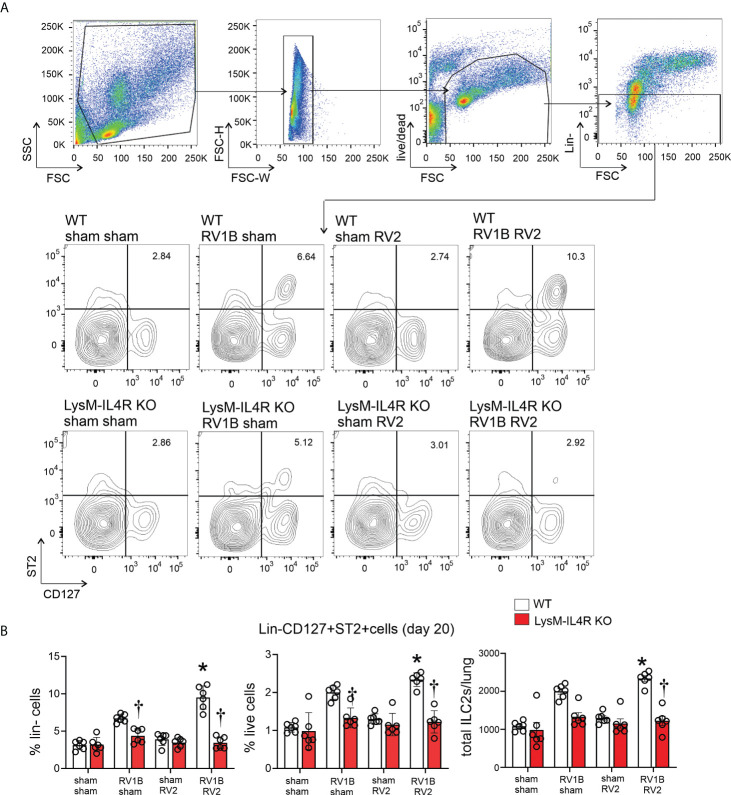
LysM^Cre^ IL-4Rα KO blocks lung ILC2 expansion in 6-day old mice following heterologous RV infection. Wild-type and LysM^Cre^ IL-4Rα KO mice were inoculated with sham or RV-A1B on day 6 of life and sham or RV-A2 on day 13 of life. On day 20, lungs were harvested, digested with liberase TM, collagenase XI, hyaluronidase 1a, and DNase I, and stained with lineage antibody cocktail, anti-CD127, anti-ST2 and Pacific blue (for dead cells). Cells were washed, fixed, and processed for flow cytometry. **(A)** Figure showing flow cytometry analysis of live lineage-negative, CD127+ ST2+ ILC2s from sham + sham, RV-A1B + sham, sham + RV-A2 and RV-A1B + RV-A2 groups. **(B)** Graph showing group mean data for ILC2s as a percentage of lineage-negative cells (left panel), as a percentage of live lung cells (middle panel) and as a total ILC2s number per lung (right panel) (N=6 from two different experiments, mean± SD; *different from wild-type RV-A1B+sham, P < 0.05 by one-way ANOVA; †different from wild-type RV-A1B + sham or RV-A1B + RV-A2, P < 0.05 by one-way ANOVA.

We next determined ILC2 number by flow cytometry at 20 days of life (14 days after primary infection either RV-A1B or sham, and 7 days after secondary infection with either RV-A2 or sham). ILC2s were identified by lineage-negative, ST2+, CD127+ cells by flow cytometry (**Figure 4A**). Wild-type mice infected with RV-A1B showed expansion of ILC2s compared to sham-infected mice, and mice undergoing heterologous infection with RV-A1B and RV-A2 showed significantly higher number of lung ILC2s (**Figure 4B**). In contrast, LysM^Cre^ IL-4Rα KO mice failed to show an increase in ILC2s, either after single infection with RV-A1B or after heterologous infection with RV-A1B and RV-A2. These data suggest that M2a macrophages promote ILC2 expansion two weeks after initial infection with RV-A1B, and after heterologous infection with RV-A2.

### Effects of the IL-4Rα KO on mucous metaplasia

**Figure 5:**
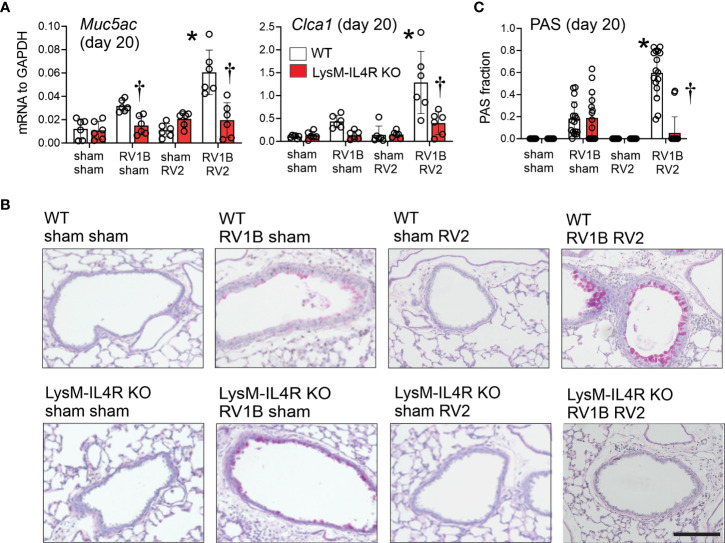
LysM^Cre^ IL-4Rα KO attenuated heterologous RV infection-exaggerated mucus metaplasia and type 2 immune responses. Six-day old immature wild-type and LysM^Cre^ IL-4Rα KO mice were inoculated with sham or RV-A1B on day 6 and sham or RV-A2 on day 13 of life. Lungs were harvested on seven days after the secondary infection (sham or RV-A2) and processed for qPCR and histology. Lungs sections were stained for PAS and quantified using NIH ImageJ. **(A)** mRNA expression of Muc5ac, and Gob5 (Clca1) in sham + sham, sham + RV-A1B, sham + RV-A2, and RV-A1B + RV-A2-infected mice. (N =6 from two different experiments, mean± SD; *different from wild-type RV-A1B + sham, P < 0.05 by one-way ANOVA; †different from wild-type RV-A1B + sham or RV-A1B + RV-A2, P < 0.05 by one-way ANOVA. **(B)** PAS staining in sham, RV-A1B, RV-A2, RV-A1B+RV-A2-infected wild-type and LysM^Cre^ IL-4Rα KO mice. The black bar is 50 microns (μ). **(C)** Quantification of PAS staining in the airways. Data are represented are PAS-positive cells per micron of basement membrane length. Data shown are mean ± SD; n=2-3 airways/mouse, 6 mice per group from two different experiments; *different from wild-type RV-A1B + sham, *P* < 0.05 by one-way ANOVA; †different from wild-type RV-A1B + RV-A2, P < 0.05 by one-way ANOVA.

Consistent with previous findings, heterologous RV infection of wild-type mice exaggerated mRNA expression of Muc5ac and Gob5 compared to mice infected with RV-A1B alone (**Figure 5A**). A similar pattern was observed when lungs were stained with PAS (**Figure 5B**). LysM^Cre^ IL-4Rα KO mice infected with RV-A1B and RV-A2 showed significantly reduced mucin gene expression and PAS staining, but mice infected with RV-A1B alone were unaffected by the KO.

### Recombinant IL-33 restores heterologous RV-enhanced mucus metaplasia and type 2 immune responses in LysM^Cre^ IL-4Rα KO mice

Our previous studies showed that, following RV-A1B infection, neutralizing antibody against IL-33 blocked RV-induced IL-25 and TSLP expression (9). Administration of recombinant IL-33 was also sufficient for type 2 inflammation and mucous metaplasia in these mice. Therefore, we tested whether intranasal administration of mouse recombinant IL-33 “rescued” the enhanced type 2 inflammation and mucous metaplasia in double-infected LysM^Cre^ IL-4Rα KO mice.

**Figure 6:**
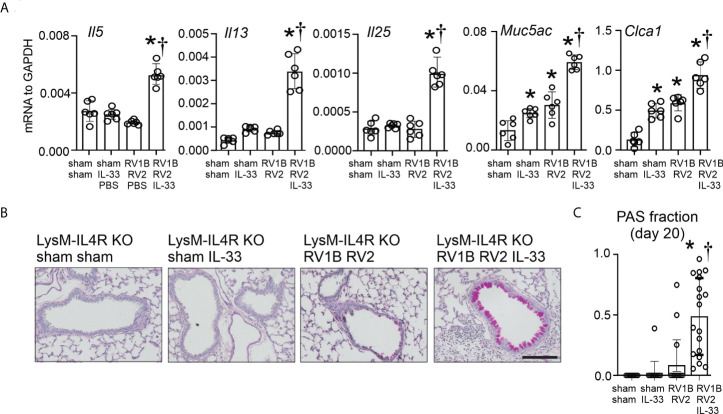
Recombinant IL-33 restores type 2 immune responses and mucus metaplasia in LysM^Cre^ IL-4Rα KO mice with heterologous RV infection. Six-day old immature LysM^Cre^ IL-4Rα KO mice were inoculated with either sham on day 6 and 13 or RV-A1B on day 6 and RV-A2 on day 13 of life. Selected LysM^Cre^ IL-4Rα KO mice were also treated with 0.1 µg of mouse recombinant IL-33 intranasally on day 13 of life. Lungs were harvested on day 20 (7 days post secondary sham or RV-A2 infection) and processed for qPCR **(A)** and histology **(C)**. **(A)** mRNA expression of IL-5, IL-13, IL-25, Muc5ac, and Gob5 (Clca1); N =6 from two different experiments, mean± SD; *different from LysM^Cre^ IL-4Rα KO sham + sham, P < 0.05 by one-way ANOVA; †different from LysM^Cre^ IL-4Rα KO RV-A1B+RV-A2, P < 0.05 by one-way ANOVA. **(B)** PAS staining in LysM^Cre^ IL-4Rα KO mice. The black bar is 50 microns. **(C)** Quantification of PAS staining in the airways. Data are represented are PAS-positive cells per micron of basement membrane length. Data shown are mean ± SD; n=2-3 airways/mouse, 6 mice per group from two different experiments; *different from LysM^Cre^ IL-4Rα KO sham+sham, P < 0.05 by one-way ANOVA; †different from LysM^Cre^ IL-4Rα KO RV-A1B+RV-A2, P < 0.05 by one-way ANOVA.

LysM^Cre^ IL4Rα KO mice were infected with RV-A1B on day 6 of live followed with RV-A2 on day 13 of life, with or without recombinant IL-33. Again, compared to sham-treated mice, heterologous RV infection of LysM^Cre^ IL4Rα KO mice failed to induce mRNA expression of IL-5, IL-13 or IL-25 (**Figure 6A**). There was a small increase in mRNA levels of Muc5ac and Gob5 (**Figure 6A**). Addition of recombinant IL-33 significantly increased mRNA levels of IL-5, IL-13, IL-25, Muc5ac and Gob5 in RV-A1B- and RV-A2-infected LysM^Cre^ IL-4Rα KO mice (**Figure 6A**). Finally, adding of addition of recombinant IL-33 rescued mucous metaplasia in LysM^Cre^ IL-4Rα KO mice with heterologous RV infection (**Figures 6B, C**).

### LysM^Cre^ IL-4Rα KO impaired epithelial derived IL-33 expression following heterologous RV infection

**Figure 7:**
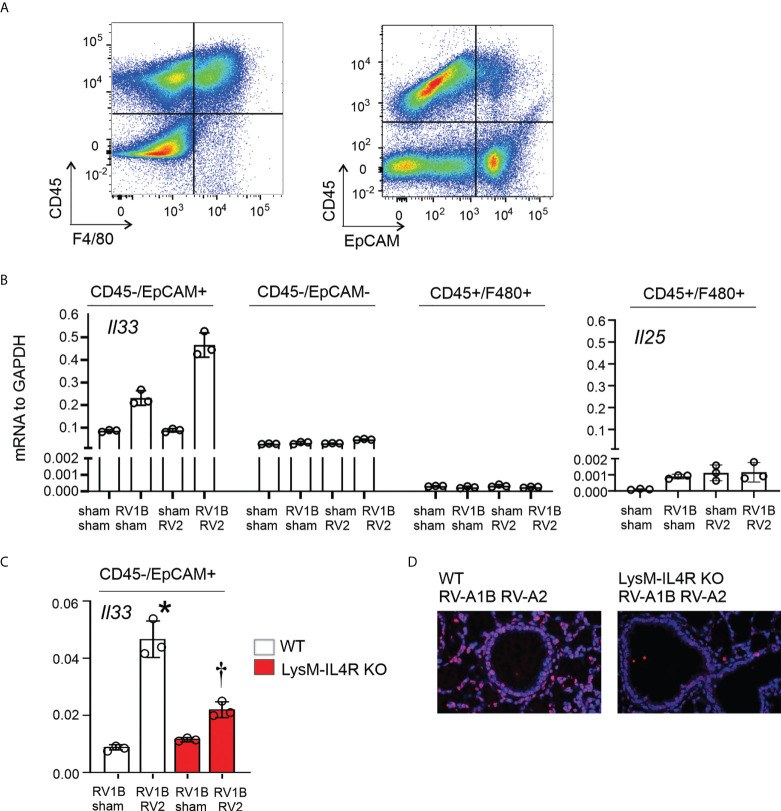
LysM^Cre^ IL-4Rα KO attenuated epithelial IL-33 expression after heterologous RV infection. Six-day old immature wild-type and LysM^Cre^ IL-4Rα KO mice were inoculated with sham or RV-A1B on day 6 and sham or RV-A2 on day 13 of life. Lungs were harvested on day 14 (1 day post RV-A2 infection) and digested with liberase TM, collagenase XI, hyaluronidase 1a, and DNase I, and stained with anti-F4/80, anti-CD45, anti-EpCAM, and DAPI (for dead cells). **(A)** Cell suspensions were sorted for CD45+F4/80+ macrophages, CD45-EpCAM+ epithelial cells, and CD45-EpCAM- cells. **(B)** The cell pellet was collected for IL-33 or IL-25 mRNA expression by quantitative PCR. IL-33 and IL-25 mRNA expression of CD45+F4/80+ macrophages, CD45-EpCAM+ epithelial cells, and CD45-EpCAM- cells in wild-type mice. (N=6/group, lungs from two mice were combined for each measurement). Data shown are mean ± SD. **(C)** Epithelial derived IL-33 mRNA expression in CD45- EpCAM+ epithelial cells in wild-type and LysM^Cre^ IL-4Rα KO mice. Data shown are mean ± SD, *different from wild-type RV-A1B + sham, P < 0.05 by one-way ANOVA; †different from wild-type RV-A1B + + RV-A2, P < 0.05 by one-way ANOVA. **(D)** Lung IL-33 in wild-type and LysM^Cre^ IL-4Rα KO mice. .

Having shown that M2a macrophages were required for enhanced type 2 inflammation and mucous metaplasia following heterologous RV infection, and that IL-33 is sufficient to “rescue” the enhanced phenotype in LysM^Cre^ IL-4Rα KO mice, we examined the cellular source of IL-33. Wild-type C57BL/6J mice were treated on day 6 of life with RV-A1B (or sham) and day 13 of life with RV-A2 (or sham). Mice were sacrificed and lungs were harvested at day 14 of age (8 days after primary infection with RV-A1B and 1 day post-secondary RV-A2 or sham infection). Cell suspensions were sorted for CD45+F4/80+ macrophages, CD45-EpCAM+ epithelial cells, and CD45-EpCAM- cells (**Figure 7A**). IL-33 mRNA was mostly detected in CD45-/EpCAM+ epithelial cells (**Figure 7B**). Within the CD45-EpCAM+ cells, IL-33 mRNA levels were highly increased in the lungs of mice undergoing heterologous infection (**Figure 7B**). Heterologous infection did not increase IL-33 mRNA levels in CD45-EpCAM- cells or CD45+F4/80+ macrophages. Next, we compared heterologous RV infection-induced IL-33 mRNA levels in CD45-EpCAM+ epithelial cells from wild-type and LysM^Cre^ IL-4Rα KO mice (**Figure 7C**). As shown in 7B, compared to RV-A1B infection alone, IL-33 mRNA levels were significantly increased in lung epithelial cells from mice undergoing heterologous infection. However, IL-33 mRNA was significantly reduced in CD45-EpCAM+ epithelial cells from heterologous RV-infected LysM^Cre^ IL-4Rα KO mice compared to wild-type mice (**Figure 7C**). Similar results were found in lung sections stained for IL-33 (**Figure 7D**).

Finally, CD45+F4/80+ cells showed modest mRNA expression of IL-25 at 14 days of age (**Figure 7B**), but the pattern of IL-25 mRNA expression did not match the pattern of type 2 inflammation and mucous metaplasia in the mice. Taken together, these results suggested that the M2a macrophage is required for IL-33 expression during early-life heterologous RV infection.

## Discussion

To examine the effect of early-life respiratory viral infection on airway inflammation, we developed a mouse model of early-life RV infection. RV infection of 6 day-old BALB/c mice, but not mature mice, induces type 2 cytokine expression, mucous metaplasia and airways hyperresponsiveness (5) which is associated with ILC2 expansion and dependent on IL-25, TSLP, and IL-33 (6, 9). Successive infection of immature mice with RV-A1B and RV-A2 produces an intensified muco-inflammatory phenotype characterized by type 2 cytokine production, increased ILC2s and mucous metaplasia. Using *Rorα* ^fl/fl^ *Il7r* ^Cre^ mice, we showed this phenotype to be ILC2 dependent (10). Recently, researchers in the field have found a reciprocal relationship between IL-13-producing ILC2s and M2a alternatively-activated macrophages, with IL-13 production by ILC2s activating M2a macrophages (16, 17), and IL-33 production by lung macrophages activating ILC2s (18–20). We therefore hypothesized that early-life RV infection polarizes airway macrophages to M2a macrophages which are in turn required for the exaggerated inflammation and mucous metaplasia in response to a second, heterologous RV infection. To test this hypothesis, we employed a transgenic mouse with LysM-specific deletion of the IL-4 receptor-α gene. LysM^Cre^ IL4Rα KO mice undergoing heterologous RV infection showed a signifcant reduction in both type 2 and innate cytokines, as well as ILC2s. IL-13-dependent expression of mucus-related genes and mucous metaplasia, as measured by PAS staining, were also significantly decreased in the LysM^Cre^ IL4Rα KO mice. These data show that M2a-like macrophages are required for exaggerated muco-inflammatory phenotype following heterologous RV infection.

In the original description of the IL-4Rα KO mice employed here, analysis of mesenteric lymph nodes by flow cytometry demonstrated reduced IL-4Rα cell surface expression in neutrophil and macrophage populations but not lymphocytes or dendritic cells (21). Further, IL-4- and IL-13-mediated cellular functions in peritoneal macrophages and non-adherent peritoneal cells (>90% neutrophils) were blocked in LysM^Cre^ IL4Rα KO mice, but T cell and bone marrow-derived dendritic cell responses were not. Since the muco-inflammatory phenotype in immature mice is not dependent on neutrophil function, we conclude that reduced inflammatory responses in LysM^Cre^ IL4Rα KO mice we observed are attributable to the loss of M2a-like macrophages. A comparative analysis of the efficiency and specificity of myeloid-Cre deleting strains using ROSA-EYFP reporter mice showed similar results, with strong YFP expression in macrophages, monocytes and neutrophils but not dendritic cell populations (24). A subsequent study using various fluorescent reporter lines showed expression in neutrophils, macrophages, dendritic cells and a subpopulation of alveolar epithelial cells (25).

M1 macrophages are classically associated with IFN-γ- or lipopolysaccharide stimulation and pro-inflammatory type 1 cytokine expression, whereas M2a macrophages are classically associated with IL-4, IL-13- or IL-33-stimulation and production of anti-inflammatory type 2 cytokines. While the M1-M2 paradigm likely represents an extreme of macrophage phenotypes (26), virus-infected macrophages are usually polarized towards an M1-like proinflammatory phenotype in the early stages of infection. However, long-term activation of lung macrophages may induce M2 polarization. For example, chronic infection of adult mice with a mouse parainfluenza virus leads to NKT cell- and IL-13-dependent alternative activation of macrophages which is first detected 21 days after infection (27). In our study, lung macrophages from mice infected with RV showed increased *Arg1* and *Retnla* expression, indicative of M2a-like polarization. Such polarization was likely a consequence of increased lung IL-4, IL-13 and IL-33 in RV-A1B-infected mice.

We then turned our attention to the mechanism by which M2a macrophages promote type 2 inflammation and mucous metaplasia. Our data suggest that lung macrophages, M2a-polarized after the first RV infection, promote airway IL-33 production following the second RV infection, thereby contributing to enhanced ILC2-dependent type 2 cytokine expression and mucous metaplasia. LysM^Cre^ IL4Rα KO mice infected on day 6 of life did not show reduced cytokine expression, consistent with the notion that M2a-like macrophages do not initiate the exaggerated asthma phenotype, but instead are responsive to type 2 cytokine expression by ILC2s. Cooperation between M2a macrophages and ILC2s after repeated RV infections could play a role in childhood asthma development.

Influenza and RSV infection each stimulate IL-33 production from lung macrophages, which in turn activates ILC2s (18–20). In this study, heterologous RV infection did not stimulate IL-33 mRNA expression in CD45+F4/80+ cells; instead, infection stimulated epithelial cell mRNA expression of IL-33 which is dependent on M2a macrophages. This pathway likely contributes to epithelial repair following viral infection. For example, after naphthalene injury, M2a macrophages are essential for bronchial re-epithelialization (28). Further studies examining the mechanism by which M2-like macrophages stimulate epithelial cell IL-33 production are warranted.

There are limitations to this study. We did not measure IL-25 and IL-33 protein production by M2 macrophages, only mRNA expression. Also, it is conceivable that M2 macrophages contribute to the asthma phenotype not only by stimulating epithelial cell IL-33 expression, but also by producing IL-13. We did not test how long mice maintain goblet cell metaplasia following the second RV infection. Finally, we did not thoroughly explore the effects of heterologous infection and LysM^Cre^ IL-4R KO on viral copy number. Compared to sham infection, infection with RV-A1B on day 6 of life significantly reduced the viral copy number observed after RV-A2 infection, a possible example of viral interference (29). LysM^Cre^ IL-4R KO mice showed a further reduction in viral copy number, consistent with the role of M1 macrophages in viral clearance.

Early-life wheezing-associated respiratory tract infection by RV is considered a risk factor for asthma development (1–3, 30, 31). Children are infected with many different RV strains, with infants having 6-10 distinct RV infections per year (4). RV infections do not induce specific immunity to reinfection by heterologous serotypes, even if viruses are from the same species (13, 14). Recurrent RV infections could result in greater degrees of airway inflammation and the potential for airway remodeling and loss of lung function over time. Our data demonstrate that successive RV infections can result in greater degrees of inflammation and mucus production than a single infection, and that M2 macrophages, along with ILC2s, contribute to this phenotype.

## Data availability statement

The original contributions presented in the study are included in the article/supplementary material. Further inquiries can be directed to the corresponding author.

## Ethics statement

The animal study was reviewed and approved by Institutional Review Board of the University of Michigan Medical School.

## Author contributions

MHa performed experiments, analyzed the data, and drafted the manuscript. HB performed experiments. SK performed experiments. SS performed experiments. AG performed experiments. YL performed experiments and analyzed data. JK performed experiments. JB performed experiments, analyzed data, and edited the manuscript. MHe supervised all aspects of the project, interpreted the data, and wrote the final draft of the manuscript. All authors contributed to the article and approved the submitted version.

## Funding

This work was supported NIH grants R01 HL134369 and R01 AI155444 (to MBH).

## Acknowledgments

The authors thank Prof. Frank Brombacher, International Centre for Genetic Engineering and Biotechnology, Cape Town, South Africa, for providing (LysM^Cre^ IL-4Rα KO) mice.

## Conflict of interest

The authors declare that the research was conducted in the absence of any commercial or financial relationships that could be construed as a potential conflict of interest.

## Publisher’s note

All claims expressed in this article are solely those of the authors and do not necessarily represent those of their affiliated organizations, or those of the publisher, the editors and the reviewers. Any product that may be evaluated in this article, or claim that may be made by its manufacturer, is not guaranteed or endorsed by the publisher.
